# A Simple Method for Encapsulating Single Cells in Alginate Microspheres Allows for Direct PCR and Whole Genome Amplification

**DOI:** 10.1371/journal.pone.0117738

**Published:** 2015-02-17

**Authors:** Saharnaz Bigdeli, Roger O. Dettloff, Curtis W. Frank, Ronald W. Davis, Laurel D. Crosby

**Affiliations:** 1 Stanford Genome Technology Center, Department of Biochemistry, Stanford University, 3165 Porter Drive, Palo Alto, CA 94304, United States of America; 2 Department of Chemical Engineering, Stanford University, Stanford, CA, United States of America; 3 Caerus Molecular Diagnostics, Mountain View, CA, United States of America; Oak Ridge National Laboratory, UNITED STATES

## Abstract

Microdroplets are an effective platform for segregating individual cells and amplifying DNA. However, a key challenge is to recover the contents of individual droplets for downstream analysis. This paper offers a method for embedding cells in alginate microspheres and performing multiple serial operations on the isolated cells. *Rhodobacter sphaeroides* cells were diluted in alginate polymer and sprayed into microdroplets using a fingertip aerosol sprayer. The encapsulated cells were lysed and subjected either to conventional PCR, or whole genome amplification using either multiple displacement amplification (MDA) or a two-step PCR protocol. Microscopic examination after PCR showed that the lumen of the occupied microspheres contained fluorescently stained DNA product, but multiple displacement amplification with phi29 produced only a small number of polymerase colonies. The 2-step WGA protocol was successful in generating fluorescent material, and quantitative PCR from DNA extracted from aliquots of microspheres suggested that the copy number inside the microspheres was amplified up to 3 orders of magnitude. Microspheres containing fluorescent material were sorted by a dilution series and screened with a fluorescent plate reader to identify single microspheres. The DNA was extracted from individual isolates, re-amplified with full-length sequencing adapters, and then a single isolate was sequenced using the Illumina MiSeq platform. After filtering the reads, the only sequences that collectively matched a genome in the NCBI nucleotide database belonged to *R. sphaeroides*. This demonstrated that sequencing-ready DNA could be generated from the contents of a single microsphere without culturing. However, the 2-step WGA strategy showed limitations in terms of low genome coverage and an uneven frequency distribution of reads across the genome. This paper offers a simple method for embedding cells in alginate microspheres and performing PCR on isolated cells in common bulk reactions, although further work must be done to improve the amplification coverage of single genomes.

## Introduction

In order to study the genetic variation between different cells in a complex mixture, one needs the ability to isolate and sequence the genomes individually. Fluorescence activated cell sorting (FACS) followed by multiple displacement amplification (MDA) is the current standard for studying single cells, where cells are separated in an aerosol stream and sorted into trays of individual wells for downstream amplification and sequencing. Processing samples for whole genome sequencing involves multiple steps, including cell lysis, DNA amplification with random primers, sample purification, and addition of new sequencing adapters for the sequencing library. However, processing cells in individual wells increases the costs associated with reagents, consumables, and high-throughput liquid handling. The goal of this project was to develop a method for isolating single cells and directly preparing DNA libraries in common bulk reactions, and then sorting later. Such a method could improve the process for generating sequencing-ready DNA from many individually isolated cells.

**Fig 1:**
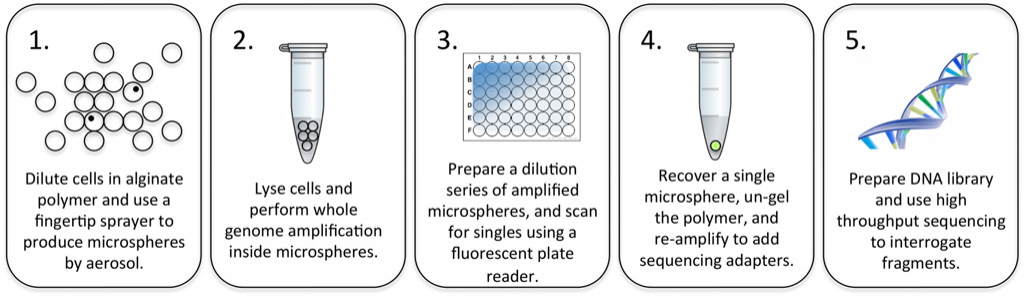
Illustration of the process workflow. 1.) Cells are diluted in alginate polymer to a concentration of approximately 10^5^ cells per microliter, resulting in a 10% occupancy rate in 100 μm microspheres. 2.) Cells are lysed using heat, and the bulk microsphere solids are mixed with reagents for a 2-step whole genome amplification reaction. 3.) After amplification, microspheres are diluted to extinction in a 384 well plate, and scanned for presence of single microspheres that fluoresce with PicoGreen DNA stain. 4.) An isolated microsphere is transferred to a fresh tube and the DNA products are recovered by dissolving the alginate matrix. These amplified products are submitted to further rounds of amplification to add sequencing adapters. 5.) The products are prepared for high throughput sequencing using the Illumina MiSeq platform. Intermediate steps include fluorescence microscopy and quantitative PCR for quality control.

The initial concept was to encapsulate cells in microfluidic droplets or water-in-oil emulsions, but commercially available microfluidic and emulsion platforms are poorly suited for isolation and recovery of individual droplets. In 2009, Walser *et al*. reported a protocol for embedding and culturing *E. coli* clones in alginate microspheres, followed by PCR and large-particle (COPAS) flow sorting [1]. Until this publication, it was generally believed that alginate inhibits PCR [2], although alginate has been used as biomaterial for cell encapsulation for well over 30 years [3]. The value of screening *E. coli* clones in alginate microspheres may not have been fully realized due to the instrumentation costs associated with sorting large particles, and advancements in competing technologies such as direct high-throughput sequencing and metagenomics. However, encapsulating single cells in alginate microcarriers offers the possibility of keeping genomes segregated while preparing the samples in common bulk reactions, and then sorting downstream. Thus, the goal of this project was to simplify the process of generating and sorting alginate microspheres, and to test the feasibility of single cell isolation and whole genome amplification in alginate microspheres. The entire process workflow is outlined in Fig. 1.

## Materials and Methods

### Materials

Sodium alginate (#71238; 70:30 ratio of guluronic:mannuronic acid) and BaCl_2_ (#B0750) were purchased from Sigma. *Escherichia coli* K12 strain DH5α was obtained from Invitrogen, and *Rhodobacter sphaeroides* 55304 was purchased from ATCC. *Rhodobacter sphaeroides* was chosen as a positive control because it has a fully sequenced reference genome, is capable of aerobic growth in the laboratory, and is a Gram negative organism for straightforward lysis. Failsafe Premix F master mix (Epicenter) was used for PCR, q-PCR, and whole genome amplification. Primers were designed and purchased from Integrated DNA Technologies, Inc. For microsphere synthesis, frosted 8 ml bottles with fingertip sprayers were purchased from Elements Bath and Body (http://elementsbathandbody.com.) Minisart NML Syringe Filters 17593 (1.2 μm) were used to filter alginate, whereas 70 μm and 100 μm cell strainers were obtained from BD Biosciences in order to fractionate the desired sizes of microspheres. Pre-sterilized Rainin LTS wide bore and narrow bore pipette tips were used for transferring microsphere suspensions, or for removing supernatants, respectively.

### Microbial culture

Stocks of *Escherichia coli* and *Rhodobacter sphaeroides* were streaked on LB agar and serially plated for at least 2 passages to obtain well-isolated colonies. For liquid culture, a single colony was inoculated in LB broth and incubated at 37°C for 24 hours for *E. coli*, and at 30°C for 4 days for *R. sphaeroides*. The use of *R. sphaeroides* provided a means of determining the extent of nucleic acid contamination during amplification and sequencing, since *E. coli* is often used as a vector for producing commercial polymerases.

### Sodium alginate preparation

Powdered sodium alginate (#71238, Sigma) was used to make 1.5% solution in sterile filtered water and heated at 65°C for 4 hours to facilitate dissolution. Stock solutions of gel were pasteurized at 100°C for 30 minutes, and stored at 4°C until further use. Immediately prior to microsphere synthesis, 50 ml of gel was filtered through 1.2 μm syringe filter and UV irradiated for 10 minutes (Stratalinker 1800, distance of 1 cm from the light source, and intensity of 1500 μJ/cm^2^.)

### Microsphere synthesis

To generate microspheres, a 5 ml volume of alginate was transferred to a fingertip spray bottle, and 125 μl of 2M KCl and 20 μl 3M NaCl was added for a final concentration of 7 mM and 75 mM, respectively. One microliter of cell suspension was subsequently added to the 5ml of alginate polymer, providing a final concentration of approximately 2 × 10^5^ cells per milliliter. The bottle was capped and thoroughly mixed by gentle end-over-end rotation for 30 minutes. Microspheres were generated by using the fingertip pump to spray the sample into a 1-liter beaker containing 200 ml of 50 mM BaCl_2_ hardening buffer on a gently stirred magnetic plate. Droplets were kept in the 50 mM BaCl_2_ hardening buffer for 10 minutes and then washed twice with 0.1 mM BaCl_2_ wash buffer. Microspheres were fractioned initially with a coarse mesh sieve to remove non-uniform gel debris, and passed through 70 μm and then 100 μm BD cell strainers to isolate the desired size range. The fractionated microspheres were stored in 20 ml of 0.1 mM BaCl_2_ storage buffer at 4°C and used within 3 days.

An online worksheet was developed for estimating the occupancy rate of microspheres based on Poisson statistics [4] (S1 Text.) With an initial concentration of 2 x10^5^ cells per ml and microsphere diameter of 100 um, the occupancy rate is predicted to be 10%. Of the *occupied* microspheres, 95% are predicted to contain one cell and 5% of the microspheres contain two cells or more. A series of control reactions with *E. coli* cells were used to measure the proportion of occupied vs. empty microspheres. The loading rate was verified by fluorescence microscopy by: 1) directly visualizing the number of cells per microsphere, and 2) amplifying DNA by PCR and counting the proportion of empty vs. amplified microspheres stained with GelGreen DNA dye.

### DNA decontamination

All work was performed inside a SterilGARD Advanced III laminar flow hood that was treated with 10% bleach solution, followed by DNA Zap surface treatment and 15 minutes of UV exposure. For post-PCR handling, samples were handled in an AirClean 600 PCR workstation, which was also treated with 10% bleach, DNA Zap, and 10 minutes of UV exposure. In order to avoid cross-contamination, filtered pre-sterilized tips were used. A dedicated set of Rainin pipettes were treated with bleach and DNA Zap, and stored in the hood. Alginate was purified as described previously using UV. All plastic ware and dishware were exposed to UV in the hood prior to use, and gloves were changed frequently to avoid contamination. Commercial polymerases are prepared in biological vectors, so it is common for *E. coli* DNA to be present as trace contaminants in commercial reagents. The heat-labile dsDNAse (Arcticzymes) was tested as a means to reduce nucleic acid contamination during amplification reactions, but was ultimately rejected after a series of sequencing run failures due to insufficient product.

### Cell lysis

Bacterial cells were lysed inside the alginate microspheres using heat. One hundred microliters of microsphere suspension was transferred to a 1.5 ml Eppendorf Safe-Lock tube using a wide-bore pipette tip (Rainin LTS). After the microspheres settled at the bottom of the tube, the wash buffer was aspirated and replaced with 200 μl of sterile filtered 100 mM Tris, 0.1 mM BaCl_2_. The tube was placed inside a heat block and incubated at 98°C for 15 min.

### Polymerase Chain Reaction

Standard PCR was used as a control to test for proper formation of microspheres, and to quantify the number of microspheres that contained cells. A variety of different primer combinations were used to produce amplicons of different sizes from the single copy DNA-dependent RNA polymerase gene from *E. coli*, *rpo*C (Table 1.) The commercial reagent Epicentre Failsafe Premix F was used as the base reagent in the following mixture: 1X Failsafe Premix F, 0.5 μM of each primer, and 0.5 U of polymerase. (Failsafe Premix F does not contain sulfate, which will form a precipitate with the crosslinking barium ions and ungel the polymer.) Approximately 5–15 μl of microsphere solids was transferred in storage buffer to a 0.2 ml thin-walled PCR tube. After aspirating the storage buffer, approximately 40 μl of PCR reaction mixture was applied to the microspheres, flicked gently to mix, and cycled in a conventional PCR thermocycler using a standard cycling protocol: an initial denaturation step at 95°C for 2 minutes, 30 cycles of denaturation (95°C for 45 s), annealing (55°C for 45 s), and extension (72°C for 30 s), and a final round of extension at 72°C for 5 minutes.

**Table 1:** Primers for 2-step PCR whole genome amplification, tailing, and qPCR.

	**PCR Control Primers, *E. coli*, *rpo*C gene**
450F	5’-TATGACCAACCTGGAACGTCAGCA-3’
946F	5’-ATCACCGGTTCTAACAAGCGTCCT-3’
1019R	5’-TGACGGAAACGACCCTGTTTACCT-3’
1219R	5’-CAGCTTCTTCGCGCTCAACCATTT-3’
1893R	5’-ATAGGCGAAGCCGGTGTACATGAT-3’
	**Whole Genome Amplification and Tailing Primers**
Tag PE1	5′-CTACACGACGCTCTTCCGATCT-KKKKKKKKNN-3′
Tag PE2	5′-TGCTGAACCGCTCTTCCGATCT-KKKKKKKKNN-3′
Amp PE1	5′-CTACACGACGCTCTTCCGATCT-3’
Amp PE2	5′-TGCTGAACCGCTCTTCCGATCT-3′
Tail PE1	5’AATGATACGGCGACCACCGAGATCTACACTCTTTCCCTACACGACGCTCTTCCGATCT-3’
Tail PE2	5’AAGCAGAAGACGGCATACGAGATCGGTCTCGGCATTCCTGCTGAACCGCTCTTCCGATCT-3’
	**qPCR Taqman for *R. sphaeroides*, *rpo*C gene**
Probe	/56FAM/ACACCAAAT/ZEN/GGCCGATCGTGAACGA/3IABkFQ/
Forward	5′-AAGGAGTCGGTGATCTTCTGCGA-3′
Reverse	5′-TGTACTGCTGCTCGAACTCCTTCA-3′

### Whole Genome Amplification

**Fig 2:**
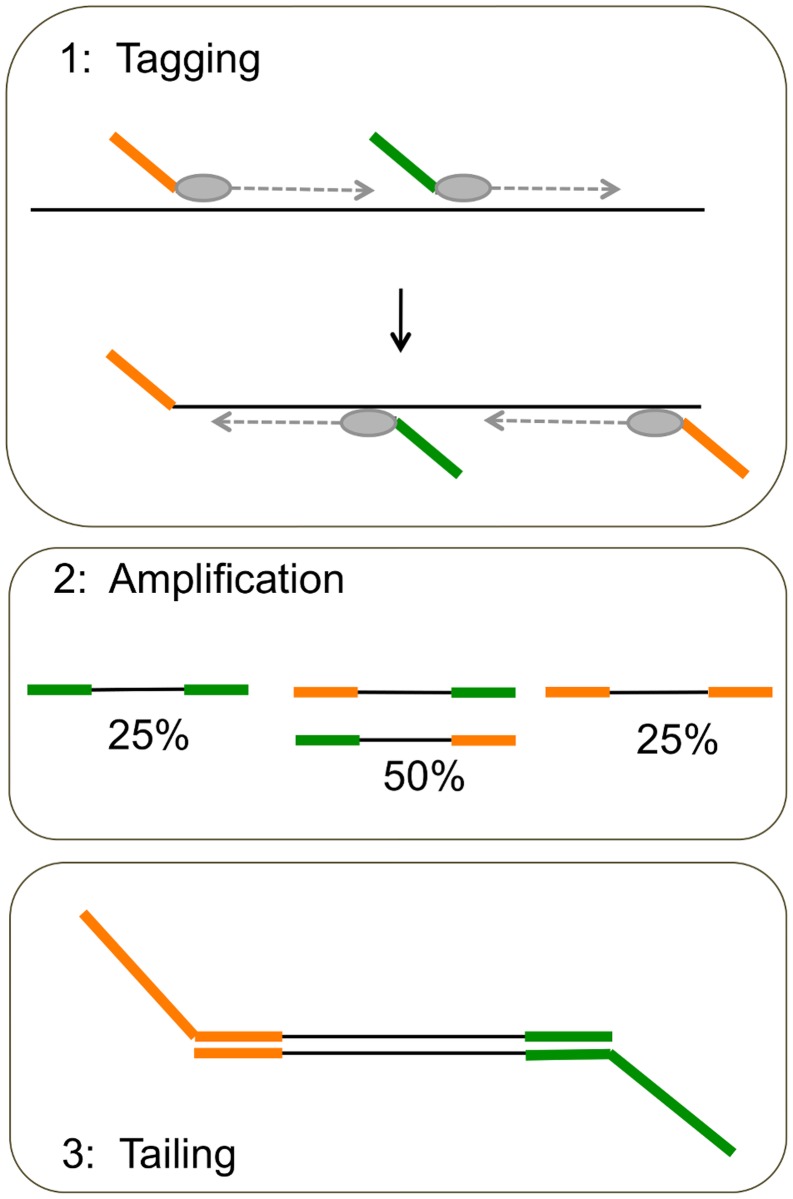
Strategy for WGA in two steps. This diagram outlines the whole genome amplification strategy in 2 steps, plus an additional step for adding the full length sequencing adapters. In Step 1, primers with 2 different tag sequences are added in a 50:50 mix. The primers anneal to the template, and a thermophilic strand-displacing enzyme (Vent exo- polymerase) is used to generate a population of fragments. For Step 2, the tag sequences are used as primers to further amplify the population of fragments. Half of the fragments are expected to have two different primer sequences on each end. After isolation of a microsphere containing fluorescent products, the sample is processed in a third reaction for addition of the full length sequencing adapters, or tails.

Multiple displacement amplification with phi29 polymerase was initially tested as a means of whole genome amplification, but consistently failed to show amplified product unless the polymerase was pre-embedded during microsphere synthesis (S2 Text.) This introduced a number of challenges for cell lysis, and the optimized protocol produced only a limited number of polymerase colonies. Thus, a modified 2-step protocol [5] was used to randomly tag the template genomic DNA with novel priming sites and to amplify from those new sites (Fig. 2.) For the first step, two primers were designed with fixed 5′ sequences that represented a portion of each of the two Illumina MiSeq paired end adapters (Table 1). On the 3′ end of each primer, a partially degenerate stretch of 8 consecutive bases of G or T was used, followed by 2 fully degenerate positions on the 3’ end (…5′- KKKKKKKKNN-3′.) This primer design was based on the commercial OmniPlex whole genome amplification kit (Rubicon Genomics.) The use of only G/T in the degenerate positions prevents formation of primer self-dimers, since self-dimers are produced exclusively when using two different 5′ tag sequences. Both tagging primers, PE1 and PE2, were combined in a 50:50 mix at a concentration of 10 μM each. A second set of primers was synthesized for the amplification reaction, which represented the same PE1 and PE2 tag sequences but lacked the randomer on the 3’ end.

*Step 1: Tagging*

The tagging reaction was prepared in a 15 μl volume with a final concentration of 1X Failsafe Premix F, 0.5 M supplemental betaine, 0.7 μM tagging primer mix, and 1.5 U Vent (exo-) polymerase enzyme (New England Biolabs.) Alginate microspheres in suspension were transferred to a 0.2 ml thin-walled PCR tube, and the storage buffer was aspirated to leave a final volume of approximately 5 μl of alginate solids. The 15 μl PCR reaction was added on top of the alginate solids and gently flicked to disperse the particles. The thermal protocol for tagging consisted of an initial denaturation step at 95°C for 2 minutes, followed by 14 cycles of denaturation (95°C for 15 s), annealing (15°C for 50 s, 25°C for 40 s, 35°C for 30 s, 65°C for 40 s), and extension (72°C for 40 s.)

*Step 2: Amplification*

The amplification reaction was prepared in a 60 μl volume with a final concentration of 1X Premix F, 0.5 M supplemental betaine, 0.7 μM Amp primer mix, and 1.5 U Platinum Taq High Fidelity polymerase (Invitrogen.) The supernatant was aspirated from the tagging reaction, and the new reaction mixture was added to the alginate microspheres and flicked gently to disperse the particles. This second PCR reaction consisted of an initial denaturation step at 95°C for 2 minutes, followed by 28 cycles of denaturation (95°C for 45 s), annealing (57°C for 45 s), and extension (72°C for 30 s.) The reaction was completed by one cycle of extension at 72°C for 5 minutes.

### Quantitative PCR

Quantitative PCR was used as a means to optimize the whole genome amplification protocols for 2-step PCR and for the addition of full-length sequencing adapters. Taqman primers were designed to target the single copy DNA-dependent RNA polymerase gene (*rpo*C) for *R. sphaeroides* (Table 1). A qPCR protocol for absolute quantification was used with a dilution of *R. sphaeroides* DNA for the standard curve. Quantitative-PCR reactions were performed in 10 μl volumes consisting of 1X Failsafe PremixF, 1 μM Taqman primer/probe mix, 1 μl of sample, 0.75 U of Taq LD polymerase (Life Technologies) and 0.2 μl of ROX reference dye (Invitrogen.) During development of the protocols for microsphere synthesis, DNA was extracted from aliquots of bulk microspheres in order to estimate changes in copy number from before and after amplification. The same qPCR protocol was also used to estimate the amount of DNA produced after sorting individual microspheres and performing tailing reactions, which provided a means of quality control prior to preparing the sequencing library.

### Fluorescence Microscopy

To verify the cell loading-rate after PCR or whole genome amplification, an aliquot of microspheres was stained with GelGreen DNA dye and visualized by microscopy. PCR supernatants were removed and microspheres were washed twice with 200 μl of 100 mM Tris, 0.1 mM BaCl_2_ and then resuspended in 200 μl of the same buffer with the addition of 1 μl of 40X GelGreen stain (final concentration 0.2X.) Microspheres were then visualized with a Zeiss Axiovert 40 CFL inverted microscope fitted with an xCite 120 series UV lamp with filter set 10 (excitation BP450–490, emission BP515–565.) The remaining unstained microspheres in the amplified sample were used for recovering single isolates with a spectrophotometer.

### Microsphere isolation by dilution series

Microspheres were manually sorted by performing a 2-fold dilution series in wells of a 384 well plate. Amplified products were stained inside the microspheres using a Quant-It Picogreen dsDNA assay kit and screened using a Victor X3 plate reader (Perkin Elmer) with excitation and emission wavelengths corresponding to those used for fluorescein detection, Picogreen protocol (0.1 s). To perform the assay, a working stock of 1 ml of Picogreen reagent was prepared by making a 200-fold dilution in TE buffer per the manufacturer’s instructions. Ten microliters of dye solution was dispensed into wells of a black ProxiPlate-384 F (Perkin Elmer). Microspheres were washed twice with 100 mM Tris, 0.1 mM BaCl_2_, and then 10 μl of the microsphere suspension was aspirated and serially diluted 2-fold in the ProxiPlate. The plate was incubated in the dark for at least 10 minutes and then scanned on a Victor X3 plate reader to determine the relative fluorescence intensity, which corresponded to total numbers of fluorescent microspheres. The contents of each microplate well were collected using a wide-bore pipette tip and the numbers of stained vs. unstained microspheres were counted manually using the fluorescence microscope.

### Reverse crosslinking by barium chelation

Barium-crosslinked microspheres are sensitive to chelation of the crosslinking ion by sulfate, and this property was used to develop a simple method for dissolving the hydrogel and recovering DNA from the microspheres. Following isolation of a single amplified microsphere, the supernatant was carefully removed and 5 μl of filtered 50 mM Na_2_SO_4_ was added to facilitate dissolution of the microsphere. The sample was incubated at 65°C for one hour, and then centrifuged briefly to concentrate the white BaSO_4_ precipitate. The supernatant containing DNA was removed and transferred to a new tube for downstream analysis.

### Sequencing library preparation

DNA products recovered from the microsphere after whole genome amplification contained partial sequences from the Illumina MiSeq paired-end adapters PE1 and PE2. To complete the full-length adapters, two 60-mers were synthesized that contained the full anchor and sequencing primers that match the respective 5’ sequences on the template. These 60-mer primers were used to add the full-length adapters in a standard PCR reaction consisting of 10–15 cycles. Hypothetically, 50% of the products would be expected to have both PE1 and PE2, and 25% for each PE1:PE1 and PE2:PE2.

Tailing-PCR was prepared in 60 μl reaction mixtures with a final concentration of 1X Failsafe Premix F, 0.5 μl of each tailing primer, 1.5 U of Platinum Taq High Fidelity enzyme, and 4 μl of supernatant from the dissolved microsphere. PCR reaction conditions for tailing included one cycle of denaturation at 95°C for 2 minutes followed by 10–15 cycles of denaturation (95°C for 45 s), annealing (60°C for 45 s), and extension (72°C for 45 s). The ideal number of cycles was determined by qPCR measurement of a single locus, which served as a coarse approximation for amplification of the entire genome.

Ampure XP magnetic beads were used to eliminate unused tailing primers in the DNA library prior to sequencing (Agencourt.) To bind the target DNA, 1.8 μl of the beads was added per 1 μl of the PCR product then placed in the magnetic stand for 10 minutes of incubation at room temperature. The supernatant containing PCR reagents and short fragments (below 100 bp) was carefully aspirated and discarded. The beads were washed twice with 70% ethanol, and dried in air for 5 minutes at room temperature. Target DNA was eluted by adding 15 μl of the elusion buffer to the beads, incubating for 10 minutes, and collecting the paramagnetic particles to the side of the tube and removing the supernatant containing the desired DNA fragments. The recovered DNA was quantified using the KK4835 DNA Library Quantification kit (for Illumina Sequencing) by KAPA Biosystems.

### Next-generation Sequencing

Sequencing was performed on the Illumina MiSeq platform, per the manufacturer’s instructions with 10 μl of a 2 nM template library, in order to generate paired end reads of 2 × 150 base pairs. Briefly, the DNA sample was diluted to 2nM using 1X TE buffer, pH 8.0. Ten microliters of the diluted sample was added to 10 μl of a freshly made 0.1 N NaOH. The mixture was briefly vortexed, spun down for 1 m, and incubated for 5 m at room temperature for full denaturation into single strands. To make a 20 pM solution of denatured DNA, 20 μl of 2 nM denatured DNA was mixed with 980 μl of the pre-chilled HT1 (provided by the manufacturer) on ice. The PhiX standard was also denatured and diluted to 8 pM in pre-chilled HT1 buffer, according to the manufacturer’s instructions. Ten microliters of the 8 pM PhiX standard was mixed with 990 μl of the 20 pM DNA library, and 600 μl of this mixture was used per run.

### Bioinformatics

Low quality sequencing reads were discarded using FASTX Toolkit (http://hannonlab.cshl.edu/fastx_toolkit/) with a quality cut-off of 90% > 30. Expected contaminants were identified using MegaBLAST [6]. A custom BLAST database was generated that consisted of phage phiX174, cloning vector pFosill-3, ILMN adapter sequences, *Homo sapiens* (GRCh37.p10), and *E. coli* str. K-12. After removing contaminant reads, the remaining reads were aligned to the *Rhodobacter sphaeroides* 2.4.1 complete genome (2 chromosomes and 4 plasmids), using a custom BLAST database and MegaBLAST. This alignment was used to make a coverage map showing the location and frequency of sequence reads along the *R. sphaeroides* genome.

## Results

### Single cell loading

**Fig 3:**
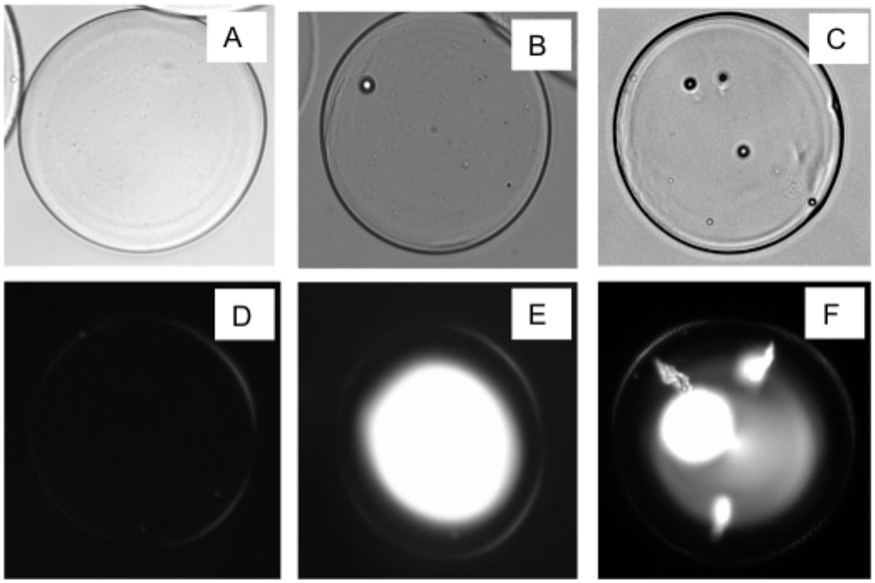
Effects of cell loading rate, visualized before and after PCR. Microscope images of 100 μm alginate microspheres containing: (A) no cells, (B) one cell per microsphere, and (C) multiple cells per microsphere. After PCR or whole genome amplification (2-step method), the DNA is stained by GelGreen and visualized by fluorescence microscopy. Images show: (D) no amplification, (E) amplification that is characteristic for one cell per microsphere, and (F) amplification of many cells, showing multiple foci of amplification and “comet tails” from cells that are trapped in the border region of the microsphere.

The concentration of microbial cells grown in liquid culture was estimated based on turbidity, and cells were diluted in alginate to a final concentration of approximately 2 × 10^5^ cells per milliliter. With microsphere volumes of 0.5 nanoliters, this predicted a loading rate where 10% of the microspheres contained at least one cell and 90% remained empty. In terms of yield, the fraction of microspheres representing the desired size range was approximately 500 μl of solids, or 10% of the original 5 ml volume of alginate. The estimated cell loading rate was confirmed by microscopic examination, since microbial cells could be clearly distinguished inside a microsphere (Fig. 3.) After PCR amplification, the interior lumen of the occupied microspheres contained fluorescent material stained with GelGreen DNA stain, whereas empty control microspheres did not fluoresce after staining. The observed ratio of fluorescent microspheres to empty microspheres was approximately 1:10. Purposefully overloading the microspheres with a higher concentration of cells led to a higher occupancy rate and as well as the production of multiple fluorescent foci after PCR.

Fluorescence microscopy helped illustrate the microsphere structure after PCR, since the stained DNA material was concentrated in the lumen of the microsphere but was not present in the boundary of the sphere. This suggests that the border and the lumen of the alginate microsphere have different structural characteristics that may influence the diffusion of DNA. Previous work has shown that barium-crosslinked microspheres are permeable to low molecular weight PCR primers, and that higher molecular weight DNA products generated *in situ* are preferentially retained [1]. This finding was replicated here for PCR and whole genome amplification from individual cells, without an *a priori* need for culturing. The observation about gel structure also led to an hypothesis that the presence of non-gelling Na^+^ and K^+^ ions in the polymer help create an inhomogeneous structure during microsphere synthesis. This hypothesis was supported by an observation that omitting the monovalent cations (Na^+^ or K^+^) from the alginate during microsphere synthesis led to PCR failure (i.e. no fluorescence.)

### Microsphere isolation

A dilution series was used in lieu of flow sorting to isolate individual microspheres using a Quant-It Picogreen dsDNA assay kit and a Victor X3 plate reader. It was observed that the microspheres tended to stack in the channel of a narrow bore pipette tip during transfer, and that surface tension could be used to dispense liquid droplets containing a single microsphere. This property helped reduce the number of dilutions needed to achieve well-isolated microspheres. Absorbance readings were determined for empty wells, wells containing one empty microsphere, and wells containing one microsphere with amplified DNA. The contents of each well were recovered and the presence of a single microsphere was validated by fluorescence microscopy. The spectrophotometer reads corresponding to each type of microsphere were based on an average of 5 samples, and are reported as followed: reagents alone (< 50 counts), one empty microsphere (50–75 counts,) and one microsphere with amplified product (85 ± 5 counts.) Thus, one amplified microsphere gave a reading that was approximately 70% greater than the value for the reagent control. In order to prevent UV damage to DNA during fluorescent microscopy, isolated microspheres that were to be used for sequencing were visually inspected by transferring the sample onto a piece of lab film (Parafilm M), aspirating the buffer, and verifying the presence of a single microsphere. For the whole genome amplification experiment, a total of 5 replicate microspheres were isolated and subjected to further processing. The replicate that ultimately produced the greatest amount of amplified product after the tailing reaction was used for a single MiSeq sequencing run.

### Measurement of WGA product by qPCR of a single locus

Quantitative PCR was used to estimate changes in genome copy number in the material extracted from a single microsphere, and to screen for the presence of free *R. sphaeroides* DNA in the dilution buffer. The buffer used to sort microspheres showed no detectable copies of *rpo*C, whereas the supernatant containing DNA from 5 replicates of a single extracted microsphere showed 10^3^ copies of the *rpo*C gene. Given that each microsphere containing fluorescent product originated from a single cell, this represents an increase of up to 3 orders of magnitude. An additional 15 cycles of PCR were used to further amplify the product after extraction and during the addition of the full-length sequencing adapters, in order to meet the minimum requirement of 20 pM DNA for the sequencing library.

### Sequencing results

DNA amplified from a single cell was recovered from an isolated alginate microsphere, and sequenced using an Illumina MiSeq. To avoid introducing contaminants, the microsphere synthesis steps and PCR preparation steps were performed inside a laminar flow hood that was treated with bleach, DNA Zap, and UV sterilization. For library preparation, Ampure beads were used to help eliminate the tailing primers that were used to add the paired ends to the template. The internal standard PhiX was added to make up approximately 50% of the total DNA in the sequencing library, as recommended by the manufacturer.

The sequencing run generated a total of 5,058,044 reads, and 34.7% were discarded as low quality reads (Table 2.) The results presented here represent the values obtained in Read 1, since a higher proportion of low quality reads were observed in Read 2. PhiX represented an average of 60% of the total reads (or 94.1% of the contaminants file), which agrees with the proportion of the internal standard that was added per the manufacturer’s instructions. Common trace contaminants such as *Escherichia coli* and *Homo sapiens* were filtered along with the internal standards, and an analysis showed that *E. coli* represented 0.3% and human sequences represented 1.5% of the sequences in the contaminants file. After filtering low quality reads and contaminants, the remaining number of reads totaled 1.9% of the total sequencing run. Of these 96,319 high quality sequences, 58.6% mapped to the *R. sphaeroides* genome. The remaining sequences not belonging to *R. sphaeroides* were used to search the NCBI nucleotide database using BLAST (with e-value = 1e-15; percent ID = 70; wordsize = 28.) The next most abundant organism called by BLAST was 524 reads of *Acinetobacter baumanii*, which represented a 100-fold lower sequence abundance compared to the 56,443 reads belonging to the *R. sphaeroides* genome.

**Table 2:** Results from Illumina MiSeq paired-end sequencing run, 2 × 150 bp.

	Read 1	%	Read 2	%
**Total number of reads**:	5058044	100%	5058044	100%
**Discarded low quality reads**: (Threshold = 90% > 30)	1756184	34.7%	1959178	38.7%
**Internal standards and contaminants**:	3205541	63.4%	3041469	60.1%
PhiX	3016505	94.1%	2855939.	93.9%
pFosill-3, sequencing adapters	130284	4.0%	127741	4.2%
*Escherichia coli*	10922	0.3%	15207	0.5%
*Homo sapiens*	47830	1.5%	42580	1.4%
**Post-filtering reads**:	96319	1.9%	57397	1.1%
*Rhodobacter sphaeroides*	56443	58.6%	31981	55.7%
*Other* (no match in NCBI database)	39876	41.4%	25416	44.2%

**Fig 4:**
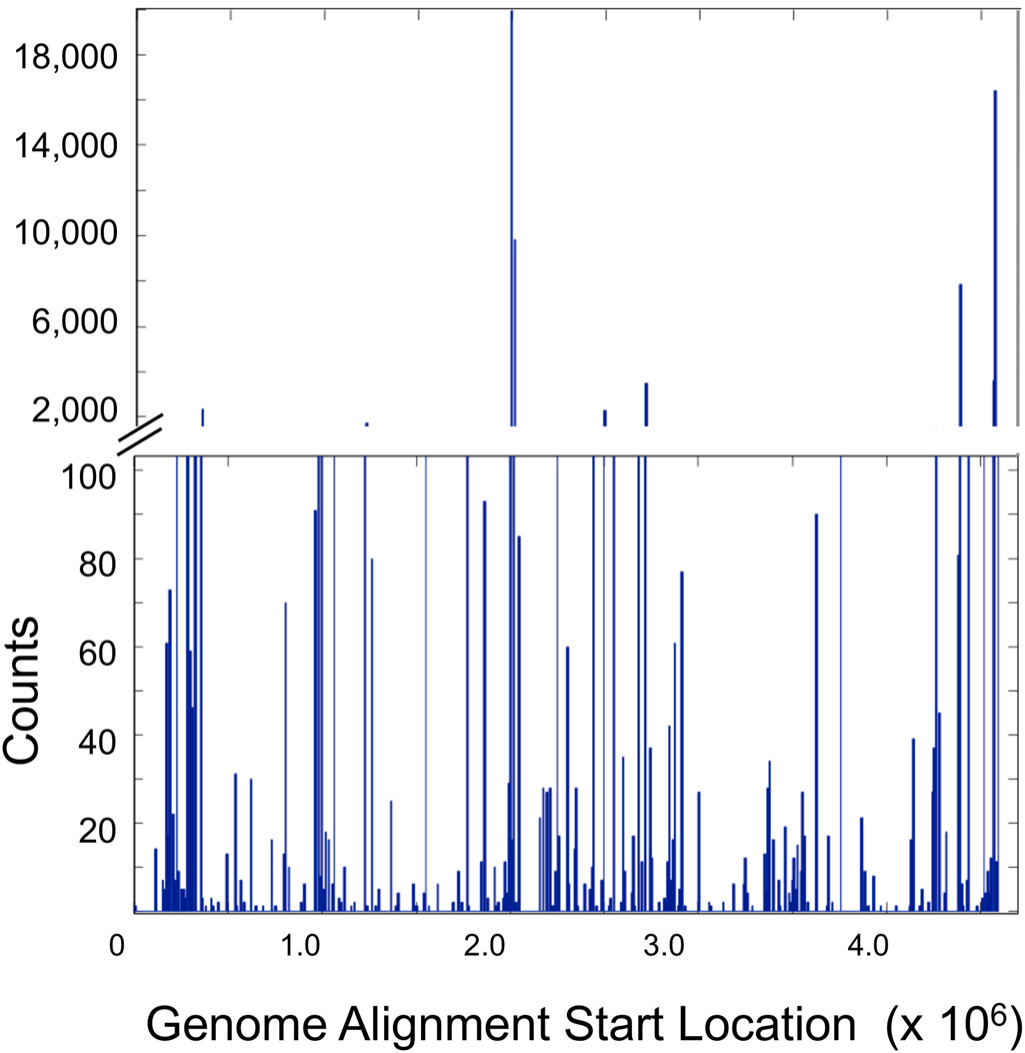
Sequence coverage of the *Rhodobacter sphaeroides* genome after whole genome amplification.

Mapping the sequence reads to the *R. sphaeroides* genome demonstrated approximately 1% coverage of the genome at 1X (Fig. 4), with 8 loci returning over 2,000X coverage. The lack of evenness is consistent with other whole genome amplification strategies from single cells [7] and reflects the problem of non-uniform priming from a single copy of a genome. The difference in genome coverage between multiple displacement amplification with phi29 (typically 30–40%) and this protocol (1%) reveals limitations of the primer design in the 2-step whole genome amplification strategy. Phi29 uses a fully degenerate hexamer primer, whereas the primer design that was adopted from the commercial OmniPlex kit by Rubicon Genomics has 8 consecutive (G/T) bases in the random portion of the tagging primer. This design helps prevent the formation of primer self-dimers that would occur exclusively with heterogeneous adapter sequences, but the base composition skews the hybridization of primers to runs of G/T in the template. This property was recognized prior to the use of this particular whole genome amplification strategy, which was chosen as a substitute for multiple displacement amplification with phi29. Accordingly, Rubicon Genomics does not promote the use of the OmniPlex kit for single cell amplification. Despite these limitations in primer design, the goal of this project was to show that multiple serial operations for whole genome amplification from a single genome could be performed, and DNA could be recovered and sequenced from a single alginate microsphere. With ongoing improvements in whole genome amplification technologies, these new strategies may be applied to cells that are isolated in alginate.

## Discussion

Alginate is a biomaterial with a long history of use for culturing cells, although the concept of using alginate microspheres as nanoliter-scale reactors for PCR is relatively new [1]. The present work suggests that the success of PCR in an alginate microsphere depends on having a loosely-crosslinked inner compartment that allows for diffusion of reagents and DNA. A number of different strategies have been proposed to achieve this structural effect, such as mixing or layering alginate with other polymers and surface coatings, or treating with chelators or alginate lyase enzyme to partially liquefy the interior of a particle [8]. Walser *et al*. used sodium and potassium salts in the alginate mixture to protect *E. coli* from osmotic stress, which is likely responsible for creating a loose structural compartment inside the microsphere that is suitable for PCR. Omitting the non-gelling ions during droplet synthesis creates a more homogeneous microsphere structure [9], which in this work led to repeated PCR failures. We propose an explanation that the presence of non-gelling ions in the polymer creates a concentration gradient between the internal environment of the droplet and the external hardening buffer. When the droplet contacts the hardening buffer, the negatively charged alginate follows the diffusion of non-gelling Na+ cations out of the droplet, thereby maintaining electroneutrality. Since barium ions are penetrating inward at the same time, this would result in a microsphere with a thick outer shell and a loose inner matrix.

Alginate may be ideal for PCR for other reasons. As a crosslinked matrix, alginate is stable under the temperature extremes in PCR and is permeable to reagents, enzymes, and low molecular weight DNA primers [1]. Higher molecular weight fractions of DNA that are polymerized *in situ* appear to be preferentially retained in alginate, possibly due to charge interactions and/or salt-bridging within the matrix [10]. These properties of the alginate microcapsule enable multiple serial operations to be performed on encapsulated genomes, including cell lysis, buffer exchanges, standard PCR or whole genome amplification, and fluorescent sorting. Alginates are simple to crosslink by external gelation with divalent cations, and can be converted into microspheres by means of droplet generators or aerosol sprays. Once hardened, the microspheres can be processed in bulk reactions and manipulated as discrete units without dilution of the amplified products. Lysis of gram negative cells inside alginate microspheres is possible using heat alone, although more robust cells may require a combination of enzymatic and physical treatments. The alginate matrix is sensitive to high salt concentrations, detergents, and chelating agents, so care must be taken to avoid these reagents during cell lysis. Finally, the crosslinks in the barium alginate can be reversed by addition of chelating agents that disrupt the microsphere and allow recovery of the DNA. The disadvantage of using barium instead of calcium as the crosslinking ion is that barium forms an insoluble precipitate with sulfate, which is often present in various commercial PCR reagents. In many cases, the sulfate is not essential and the reagents can be reformulated.

This work demonstrates a strategy for isolating single cells and generating sequencing-ready DNA from a single genome. Single cell loading was estimated based on Poisson statistics and the assumption of a well-mixed system with cells being completely dispersed in the polymer. This model also assumes that biological samples are freely dispersed (not aggregates of cells) and that the number of total cells can be estimated within an order of magnitude. There were no indications of bacterial cells settling in the medium viscosity polymer at 1.5% concentration, which is consistent with previously published work [1]. The predicted occupancy rate was also verified by microscopy both before and after PCR.

In terms of whole genome amplification and sequencing of single microbial cells embedded in an alginate matrix, the major limiting factor is compatibility with the random amplification protocol. *E. coli* contains a genome of 4 × 10^6^ base pairs, representing approximately 4000 genes and a femptogram of DNA. This quantity of DNA is six orders of magnitude less material than required for the current Illumina MiSeq platform. The standard approach for random amplification using phi29 benefits from the prolific amplification and the exceptionally high replication fidelity of this enzyme, although the hyperbranched material must be further processed to prepare the template library for sequencing. In this work, phi29 was initially tested as a means to amplify DNA inside the alginate microspheres, but the technique required the mesophilic phi29 enzyme to be pre-embedded during microsphere synthesis. This introduced new challenges for lysing cells and reformulating reagents to be compatible with alginate. A protocol for phi29 was eventually developed that generated a small number of polymerase colonies (S2 Text), but the fluorescence intensity of a polony in single microsphere would not likely have been detectable by the fluorescent plate reader. Thus, a modified 2-step strategy for tagging and amplifying DNA was used to perform whole genome amplification. Variations of this basic strategy have been used for random amplification of genomic DNA, including sample prep for viral genotyping [11], the commercial OmniPlex kit from Rubicon Genomics [12], and multiple annealing and looping-based amplification cycles (MALBAC) [13]. The alternative 2-step PCR approach for randomly tagging and amplifying the DNA showed that genetic material could be amplified inside an alginate microsphere and then recovered afterwards. This approach also has the advantage of coupling the amplification step with the incorporation of sequencing library adapters, which offers a means of simplifying the library preparation step. However, the primer design used in this study has the drawback of using only a partially degenerate set of bases (which was used to prevent self dimers), and this limited the sequencing coverage of the whole genome. Poor sequencing coverage appears to be the bottleneck in effective whole genome amplification from single cells, and multiple displacement amplification with phi29 remains the current standard.

In conclusion, this work offers a simplified strategy for encapsulating cells in alginate microspheres and recovering amplified products for downstream analysis. Further work must be done to improve the whole genome amplification protocol in order to provide more uniform coverage during whole genome amplification, and to increase the proportion of templates that have heterogeneous sequencing adapters on each end.

## Supporting Information

S1 Text Cell Loading Worksheet. An online worksheet was developed to help estimate the distribution of single cells in alginate microspheres.
(DOCX)

S2 Text WGA with Phi29. Multiple displacement amplification with Phi29 was tested as a means of whole genome amplification inside alginate microspheres.
(DOCX)
