# Supplementary material for: A Simple Method for Encapsulating Single Cells in Alginate Microspheres Allows for Direct PCR and Whole Genome Amplification
Source: PLoS One. 2015 Feb 17;10(2):e0117738. doi: 10.1371/journal.pone.0117738 (PMC4331554; doi:10.1371/journal.pone.0117738)
Supplement: S1 Text — An online worksheet was developed to help estimate the distribution of single cells in alginate microspheres. (DOCX) [file pone.0117738.s001.docx]

Supplementary Information: S1 Cell Loading Worksheet

A cell loading worksheet was constructed to help estimate the distribution of single cells in alginate microspheres. Variables can be adjusted to obtain a desired occupancy rate, or to quantify the number of microspheres generated in a run.

This worksheet has been made available online at:

<http://sequence.stanford.edu/cgi-bin/Cell_Loading_Worksheet/cell_loading.cgi>

Variables:

1. Measure the cell concentration, or estimate to the nearest order of magnitude.
2. Input the volume of alginate, in microliters.
3. If the cell stock is too concentrated, it is possible to dilute the sample by a desired factor (e.g. 10)
4. Identify how much culture is to be added.
5. An aerosol sprayer will produce a variety of different bead sizes, which can be fractionated by cell sieves with desired cutoffs. Select the largest size fraction from the range, and take the radius. (e.g. 100 um cell sieve corresponds to a 50 um bead radius or smaller.)
6. Estimate the amount of mass recovered by settling the fractionated beads in a graduated cylinder.

Results:

1. Given the concentration of cells in the stock and the volume of alginate, this value represents the final cell concentration in alginate before the generation of microspheres.
2. Bead volume is calculated using the equation: V = 4/3Πr^3^, and the result is provided in terms of both milliliters and nanoliters.
3. The number of beads produced is given in terms of beads per one milliliter of alginate. Multiply by the total number of ml of alginate to get the total number of beads produced per run.
4. To ensure that occupied microspheres contain only 1 cell at least 95% of the time, adjust the variables so that the number of empty microspheres is greater than or equal to 90%.

Example Screen Shot:

^
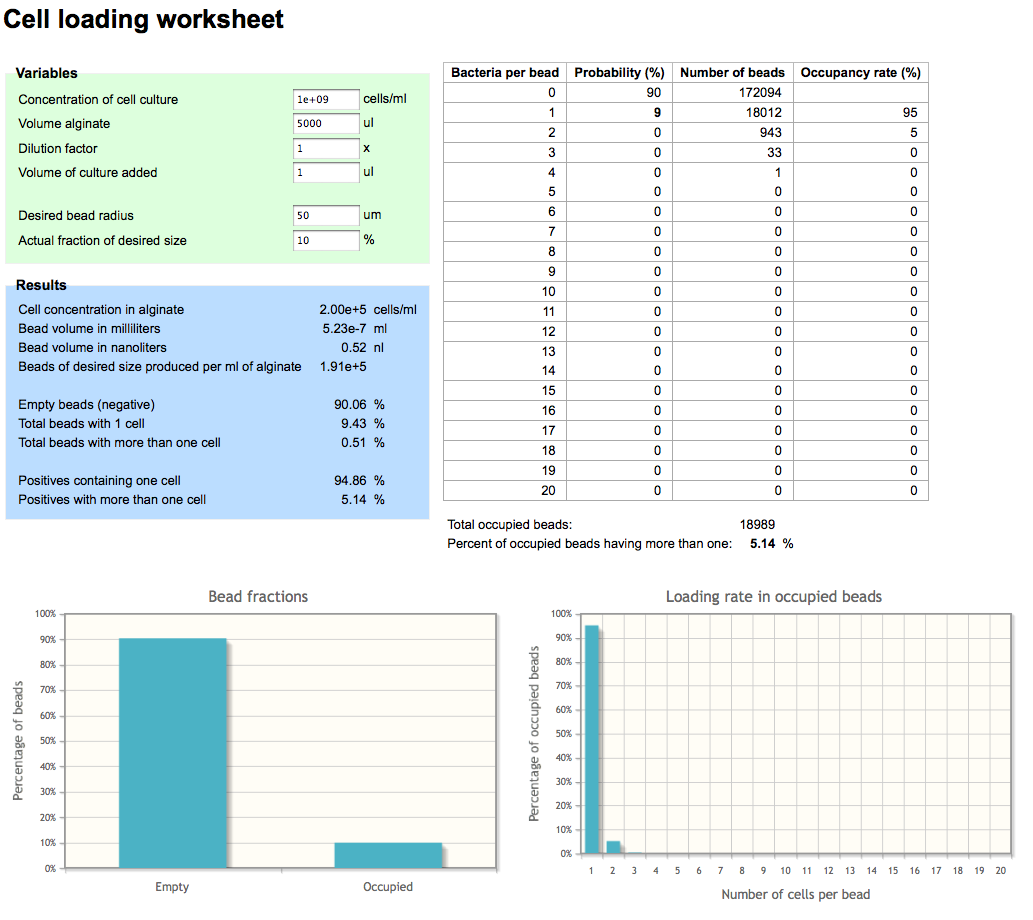
^
