# Supplementary material for: A Simple Method for Encapsulating Single Cells in Alginate Microspheres Allows for Direct PCR and Whole Genome Amplification
Source: PLoS One. 2015 Feb 17;10(2):e0117738. doi: 10.1371/journal.pone.0117738 (PMC4331554; doi:10.1371/journal.pone.0117738)
Supplement: S2 Text — Multiple displacement amplification with Phi29 was tested as a means of whole genome amplification inside alginate microspheres. (DOCX) [file pone.0117738.s002.docx]

**Whole genome amplification with phi29.** Multiple displacement amplification (MDA) with phi29 is a standard method to randomly amplify genomic DNA from single cells isolated by FACS. For this project, commercial phi29-based amplification kits could amplify DNA in the presence of alginate, but the sulfate in the buffer interfered with the integrity of the barium-crosslinked microspheres. Reformulation to a sulfate free buffer gave positive results in control reactions without alginate, but MDA amplification inside alginate microspheres was not observed. It was hypothesized that phi29 was unable to properly interact with the DNA, possibly due to an inability of the enzyme to enter the gel, or the inability of hyperbranched DNA products to diffuse and make contact with other copies of the enzyme. This hypothesis was tested by pre-embedding phi29 along with the cells prior to microsphere synthesis, although this approach precluded use of heat as a cell lysis strategy. To overcome the lysis problem, pressure cycling was used to disrupt cells inside the alginate microspheres using a Barocycler NEP2320 (Pressure Biosciences.)

The MDA base reagent included 40 mM Tris-HCl pH 8.8, 4 mM MgCl_2_, 20 mM KCl, 0.2% Triton x-100, which was amended with 1 mM dNTPs and 50 uM *oro-N6 primers. **(**The 3’-OH on the terminal and penultimate bases of the random hexamer must be protected from the 3’-5’ exonuclease activity of phi29 by *phosphoorothioate.) Cells were diluted in alginate as described in Methods, and one milliliter was transferred to a spray bottle. One hundred units (10 ul) of phi29 enzyme (New England Biolabs) was added to the alginate and gently mixed for another 10 minutes. Microspheres were synthesized by aerosol using a fingertip spray bottle. In order to lyse cells, microspheres were treated in a Barocycler using 1 min cycles alternating between 30 KPa and 5 KPa (30 cycles, 60s ON, 5s OFF.) Microspheres were processed in a PulseTube, and 0.1 mM BaCl_2_ wash buffer was used to bring the sample up to required volume. After lysis, microspheres were collected by pipette and settled in a 1.5 mL tube. A 10 ul aliquot of solids was transferred to a 0.2 ul PCR tube, the supernatant was aspirated, and the MDA reaction buffer was added to the tube. Samples were incubated at room temperature for 16 hours, and then visualized by fluorescence microscopy using GelGreen DNA stain.

**Results and Discussion**

**Whole Genome Amplification with phi29.** Multiple displacement amplification with phi29 resulted in formation of a small number of microspheres with a single tightly clustered fluorescent polymerase colony, or polony. The diameter of the polonies ranged from 6-8 μm, instead of filling the interior lumen with DNA as observed with conventional PCR. Considering that phi29 generates long strands of hyperbranched products, this suggests that DNA diffusion was limited using this whole genome amplification strategy. Although DNA was generated with phi29, the technical challenges involved with using a heat-sensitive enzyme were considered impractical for further optimization. Pre-embedding the enzyme increased the reagent costs and required specialized equipment for pressure-assisted cell lysis. In addition, the small diameter polony would likely not have generated a sufficient fluorescent signal for downstream sorting by hand using dilution series and a fluorescent plate reader. Thus, the MDA method was discarded in favor of the alternative 2-step method for whole genome amplification.


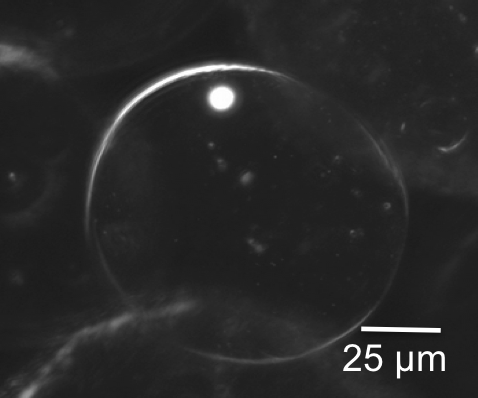


**Figure 5. Phi29 generates polymerase colonies (polonies) in alginate.**

Multiple displacement amplification in alginate required the enzyme to be added during microsphere synthesis. This prevented the use of heat for cell lysis, and also resulted in small fluorescent foci that would likely have been undetectable using the fluorescent plate reader.
